# Poisoning by Feeding on Sinapis Nigra1Read at the annual meeting of the Wisconsin Society of Veterinary Graduates.

**Published:** 1902-04

**Authors:** F. J. Roub

**Affiliations:** Monroe, Wis.


### POISONING BY FEEDING ON SINAPIS NIGRA.[1]

BY F. J. ROUB, V.S.,
MONROE, WIS.

ON November 23, 1901, at 1 P.M., I was called by 'phone, by a Mr. Lawver, living twelve miles west, to come at once; very sick cows. I informed Mr. Lawver that I could not comply with his wish before evening, as there were two calls for my services just previous to his call. He stated that if I could not come by 5 P.M., not to come at all, and very little did I care to make the visit, for I knew he was in the habit of patronizing empirics and was discourteous with qualified practitioners. After making the two calls previous to Mr. Lawver's, and having ample time to arrive at his farm by 5 P.M., I concluded to go. On my arrival, you can imagine my surprise ; in front of the barn lay ten cadavers, minus their epidermis, and one cow in the barn in the last throes of death, and seven cows that were very sick. I was informed by the owner that all of them would die, for the symptoms were in unison with the ones that died. This was the owner's prognosis, not mine. All were milk cows, and were highly fed in order to produce greater profits by a large flow of milk, and showed better care than the majority of cows receive. After taking in the surroundings and considering everything in a good hygienic condition, and being at a loss as to what the cause originated from, I asked the owner for information in regard to feeding, watering, and change of pasture. The owner stated as follows : "I stable them at night, feed clover hay with a ration of ground feed night and morning, turn them out mornings, feed shock-corn in the yard; the remainder of the time they run at large over the farm." Bear in mind, November 23d, at 5 P.M., I arrived at Mr. Lawver's farm; but the day previous, the 22d, at 3 P.M., these cows were turned into a small lot, about four acres, situated on river bottom, between the barn and river, the

[1] Read at the annual meeting of the Wisconsin Society of Veterinary Graduates.

lot being utilized to raise corn for early feed in autumn; after the corn was cut off, mustard grew up in abundance, and, as there had been no stock allowed to run on this lot previous to November 22d, the owner thought he would turn his cows in and let them pick up what rough feed there was left, the cows being in from 3 P.M. until 5 P.M., only two hours, then being housed in the barn for the night, and given their evening ration; all of them feeding normally; three hours later he noticed four of the cows being sick, the owner sending at once for an Illinois empiric. As near as I could learn, his treatment was hot teas, coffee, monkshood, Ward's liniment and painkiller. As more of the herd continued to get sick, and by midnight two had died, the doctor, if I may call him such, declined to give further treatment, departing for his home, leaving the owner alone to battle with the afflicted cows.

The cows continued to get sick one after another, and so they continued to die, at intervals, from one to two hours apart. As I stated before, this was on the 22d, and I was called on the 23d, at 1 P.M., but did not arrive at destination until 5 P.M.; ten cows were dead and the eleventh one dying, and seven more sick, and seemingly going in the same channel.

At this point I became very anxious to find the real cause, anticipating that by holding an autopsy on the two cows that died just previous to my arrival it might reveal to me the much-desired information. Proceeding with the autopsy, and dissecting through into the viscera, there was an abundance of yellow fluid in the abdominal cavity, and very strong fumes of mustard being present; speaking of this to the owner, he informed me that mustard grew up in abundance in this lot, and that he noticed the cows eating it, and they seemed to relish the same. Continuing with the autopsy, there were from three to four gallons of this yellow fluid in the abdominal cavity; bladder full and of the same color; over the walls of the rumen following the bloodvessels and lymphatics there was a yellow, gelatinous mass, varying from one to three inches in thickness; the intestinal tract showed a slight enteric condition; on opening the rumen I found from one peck to a half-bushel of mustard stalks and leaves. Satisfying my own mind that the trouble was due to mustard poisoning, I turned my attention to the seven head in the barn that were sick.

Owner's prognosis: all will die. My prognosis was zero.

Cows standing, with dull, haggard expression. Extremities cold. Some tympany; respirations labored; could be made to move only by main force; then would stagger and fall unless supported, showing that locomotion was greatly interfered with. When fatal would stand in one position without moving, until they would fall and lie in a semi-conscious condition from one to three hours before death.

*Treatment.* I ordered one and one-half pounds of sulphate of soda to each one of the sick cows, and to follow up with one pound every twelve hours, until the bowels were moved; in addition, I gave nux vomica and spirits of nitre. The seven head under this treatment made a fine recovery and regained the normal flow of milk.

### NEW PUBLICATION.

Department of Agriculture: *Seventeenth Annual Report of the Bureau of Animal Industry.* Dr. D. E. Salmon, Chief of the Bureau.

This report, covering nearly every subject of importance to the live-stock industries of our country, is a book of nearly 650 pages. It is replete with beautiful illustrations of matters of great interest to the veterinarian. The various divisions of the Bureau have their special reports and tables. The infectious and contagious diseases of animals, the meat-inspection work, the exportation of live animals, and the great advances in breeding are copiously treated of. Some thirty-seven pages are devoted to the consideration of rabies. One school fails to record a single case in twelve years. Most veterinarians have only heard of cases in the human family.

### LEGISLATION.

SENATE Bill No. 88, of the Massachusetts Legislature, creating a single Chief of Cattle Commission, has passed both Houses of the Legislature and has been signed by Governor Crane.

Kansas City is fast looming up as a veterinary medical centre. Its schools claim an attendance of about one hundred and forty students the present college year.

